# Fluoride resistance in *Streptococcus mutans*: a mini review

**DOI:** 10.1080/20002297.2017.1344509

**Published:** 2017-07-06

**Authors:** Ying Liao, Bernd W. Brandt, Jiyao Li, Wim Crielaard, Cor Van Loveren, Dong Mei Deng

**Affiliations:** ^a^ State Key Laboratory of Oral Diseases, Sichuan University, Chengdu, China; ^b^ West China College of Stomatology, Sichuan University, Chengdu, China; ^c^ Department of Preventive Dentistry, Academic Centre for Dentistry Amsterdam, University of Amsterdam and Vrije Universiteit Amsterdam, Amsterdam, The Netherlands

**Keywords:** Fluoride, *Streptococcus mutans*, antimicrobial resistance, dental caries

## Abstract

For decades, fluoride has been used extensively as an anti-caries agent. It not only protects dental hard tissue, but also inhibits bacterial growth and metabolism. The antimicrobial action of fluoride is shown in three main aspects: the acidogenicity, acidurance, and adherence to the tooth surface. To counteract the toxic effect of fluoride, oral bacteria are able to develop resistance to fluoride through either phenotypic adaptation or genotypic changes. Strains that acquire fluoride resistance through the latter route show stable resistance and can usually resist much higher fluoride levels than the corresponding wild-type strain. This review summarizes the characteristics of fluoride-resistant strains and explores the mechanisms of fluoride resistance, in particular the recent discovery of the fluoride exporters. Since the fluoride resistance of the cariogenic bacterium *Streptococcus mutans* has been studied most extensively, this review mainly discusses the findings related to this species.

## Introduction

Fluoride is the most popular caries-preventive agent. It is widely used in many oral care products, such as toothpastes, mouthwashes, and gels [1]. In addition, fluoride intake through drinking water is also very common. In 2012, about 435 million people from 25 countries received water fluoridated at the recommended concentration, while people from another 28 countries have naturally fluoride-containing water with fluoride concentrations sometimes above the recommended level [2]. It is believed that the anti-caries effects of fluoride are due to its ability to protect dental hard tissues and inhibit bacterial growth and metabolism. On the one hand, fluoride in saliva can be absorbed to the surface of apatite crystals in acidic environments, which arrests demineralization. When the pH rises, fluorhydroxyapatite becomes highly supersaturated, which enhances the remineralization process [3]. This protection of enamel can be observed at fluoride concentrations as low as 0.02 ppm (0.001 mM) [4]. On the other hand, fluoride is toxic to bacterial cells and can function as an antimicrobial, specifically at concentrations during and shortly after the use of oral hygiene products. Consecutive application of fluoride at 250–12,300 ppm (13–647 mM) has been shown to reduce the numbers of *Streptococcus mutans* in dental plaque significantly [5–7]. The antimicrobial action of fluoride is enacted in three main aspects of the metabolism of oral bacteria: the acidogenicity, acidurance, and adherence to the tooth surface [5,8,9].

Bacteria have evolved different abilities to withstand certain levels of fluoride [10]. Fluoride-resistant strains of several oral bacterial species, including *S. mutans*, *Streptococcus salivarius*, and *Streptococcus sanguinis*, have been created in laboratories [11–13]. Generally, a fluoride-resistant strain is able to grow in an environment containing 400–1,000 ppm (21.1–52.6 mM) of fluoride, depending on the strain. This level of fluoride is at least three times higher than that which fluoride-sensitive strains could withstand [13,14]. The acquired fluoride resistance can be transient or permanent. Transient resistance is quickly lost by bacterial cells, already after one to seven passages, in a fluoride-free medium [15]. It is possibly acquired through phenotypic adaptation. Several transient fluoride-resistant *S. mutans* strains have been isolated from xerostomia patients [15,16]. Stable, or permanent, fluoride resistance persists for at least 50 generations after the strain is cultivated without fluoride [13]. It is considered to be a consequence of chromosomal alterations [17,18]. The laboratory-derived fluoride-resistant strains mostly show stable fluoride resistance.

The stable fluoride-resistant strains allow researchers to examine cariogenicity and fitness of the strains and to investigate the mechanism of the acquired resistance. This knowledge might help the potential impact of fluoride on oral bacteria after 50 years of daily fluoride application at high concentrations to be better understood. Since *S. mutans* has been widely recognized for its major role in cariogenesis, fluoride resistance of this species has been most often studied [19]. This review summarizes the studies on fluoride-resistant *S. mutans* strains and provide an update to the current knowledge about the potential mechanisms behind stable fluoride resistance.

## Mode of antimicrobial action of fluoride

**Figure 1.**
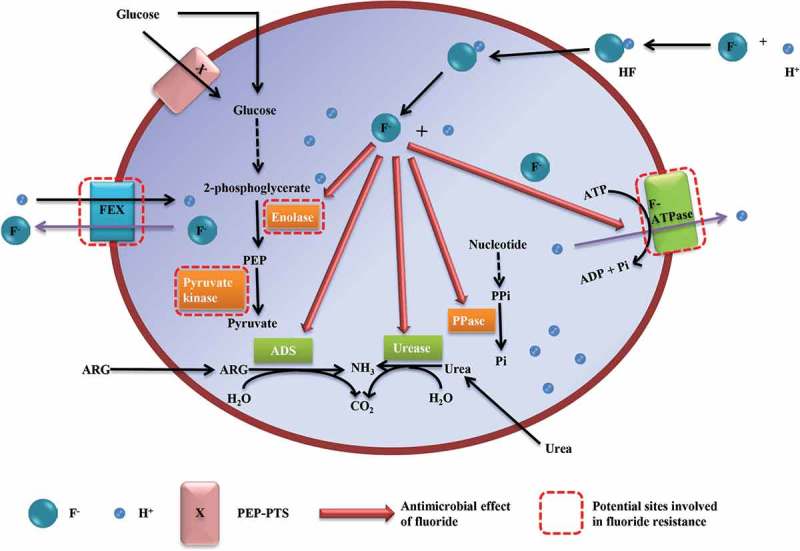
Mechanisms for the antimicrobial effects of fluoride and potential sites involved in the mechanisms of fluoride resistance. FEX, fluoride exporters; ARG, arginine; ADS, arginine dehydrolase system; PPi, inorganic pyrophosphate; PPase, pyrophosphatase; Pi, inorganic phosphate; PEP, phosphoenolpyruvate. Red arrows indicate the inhibitory effect of fluoride on the enzymes. Red dashed boxes indicate sites which are potentially involved in fluoride resistance. F-ATPase and enolase are involved in both the antimicrobial action of fluoride and the potential mechanisms of fluoride resistance.

To understand the characteristics of fluoride-resistant strains and the mechanism of acquired fluoride resistance, the mode of antimicrobial action of fluoride against *S. mutans* must be known. A brief summary of the antimicrobial mechanisms of fluoride in *S. mutans* is shown in Figure 1. The inhibitory effect of fluoride on intracellular metabolism depends on the influx of hydrogen fluoride (HF), which diffuses into bacterial cells, and dissociates to the proton (H^+^) and fluoride ion (F^–^) in the cytoplasm [20]. This process speeds up when the pH of the extracellular environment decreases, as this facilitates the association of H^+^ and F^–^ to HF [21]. Therefore, when the extracellular pH lowers, F^–^ and H^+^ accumulate faster in the cytoplasm, and the antimicrobial effect is stronger. The strong pH-dependence of the fluoride effect is well recognized. The inhibitory levels of fluoride for the glycolysis are as high as 10 mM at neutral pH but are only in the micro-molar range at pH 4.0 [21].

The intracellular F^–^ and H^+^ can directly or indirectly affect enzymatic activities and physiological processes in the cell, leading to lower acid production, acid tolerance, and adherence of *S. mutans* to tooth surfaces [5,8,9,22–25]. It has been found that enolase, which is involved in glycolysis, can be competitively inhibited by F^–^ [26]. This inhibition is observed for purified enolase as well as enolase from permeabilized cells [26–28]. In addition, enolase is also indirectly inhibited by the acidification of the cytoplasm caused by the accumulation of H^+^ [21,25]. Moreover, enolase not only plays a role in the glycolytic process, but also catalyses the production of phosphoenolpyruvate (PEP) for glucose uptake through the PEP-dependent phosphotransferase system (PTS). Thus, the inhibition of the enolase activity by F^–^ also has a negative effect on glucose uptake [9,29].

*S. mutans* experiences rapid and dynamic pH fluctuations from pH 7.0 to below pH 3.0 in the oral cavity after dietary carbohydrate intake of the host [30,31]. The ability of *S. mutans* to withstand these repetitive cycles of acid shocks is defined as acidurance or acid tolerance [30]. It is one of the major virulence factors of *S. mutans*. In the presence of fluoride, this ability has largely diminished [21,23,32]. The glycolysis of *S. mutans* stops at pH 6.0 in the presence of 10 mM F^–^, while in the absence of F^–^^,^ it is only inhibited at a pH lower than 5.0 [23]. The survival rate of *S. mutans* after exposure to a lethal pH (3.5) decreases 77% in presence of fluoride (500 mM) [32]. The acidification of the cytoplasm *via* the influx of HF, as well as the inhibition of the proton-extruding F-ATPase, accounts for the reduction in acidurance of *S. mutans* [5,23,33]. F-ATPase, also known as ATP synthase, is a membrane-bound protein consisting of two domains: F_0_ and F_1_. ATP hydrolysis by F-ATPase is obligatorily coupled to proton extrusion through the F_0_ pore in the membrane [33]. Therefore, F-ATPase is closely related to the acid tolerance of bacteria [33]. F^–^ can bind to F-ATPase in the presence of Al^3+^ [21], and the activity of the enzyme is reduced by 50% with <100 ppm (5.26 mM) fluoride [34,35]. Previous studies on fluoride inhibition of F-ATPase were done either with the purified enzyme or in permeabilized cells. The inhibition of F-ATPase in intact cells remains unknown [34–36]. It is worth mentioning that the development of modern biotechnology makes this type of study possible. The combination of single-molecule fluorescence resonance energy transfer (smFRET) and confocal microscopy allows the observation of regulatory conformational changes of specific proteins [37]. Such an approach has been taken to study the inhibition of *Escherichia coli* membrane F-ATPase by aurovertin [38] and is thus promising to confirm the fluoride inhibition of F-ATPase in *S. mutans* cells.

Fluoride can affect the adherence of *S. mutans* to enamel, which is a cariogenic trait of *S. mutans*. Yet, there is no consensus on how fluoride influences the ability to adhere [8,15,22]. While an obvious decrease in adherence of *S. mutans* to hydroxyapatite was found with 500 ppm (26.3 mM) F^–^or even less in some *in vitro* studies [8,39], others reported hardly any change in adherence with >5,000 ppm (263 mM) F^–^ [15,22]. As glucosyltransferases (GTFs) play an important role in bacterial adhesion [40], the effect of fluoride on GTF activities have been studied. Yet, no inhibition of the GTF activity by fluoride has been reported [39,41,42]. Whether the fluoride inhibition of the adherence of *S. mutans* contributes to caries prevention requires further studies.

In addition to the abovementioned actions, other mechanisms may play roles in the antimicrobial activity of fluoride. Recently, fluoride was found to inhibit alkali production [21,43]. This is due to the inhibition of either urease or the arginine deiminase system (ADS) . The former is very sensitive to fluoride, with 50% inhibition by 0.3 mM F^–^ [43,44]. The ADS, however, is less sensitive than the urease system, and its inhibition by fluoride requires low pH values [43,45]. Fluoride can also affect metabolism by binding to pyrophosphatase in the presence of Mn^2+^ [21]. Pyrophosphatase is responsible for the release of pyrophosphate (PPi) from nucleotide triphosphates and is therefore involved in a variety of physiological processes, including biosynthesis and regulation of metabolism [21,46].

## Occurrence of fluoride resistance

Laboratory-derived fluoride-resistant *S. mutans* strains have been isolated through either one-step or stepwise procedures [13,47–51]. In the one-step procedures, the wild-type cells were directly spread on agar plates containing high concentrations of NaF (highest at 26.3 mM), and fluoride-resistant colonies were selected from these plates [13,47,48]. In the stepwise procedures, fluoride-resistant strains were obtained by culturing the fluoride-sensitive parent strains on agar plates containing increasing concentrations of NaF to a maximum of 52.6 mM [50,51]. In the 1970s, *S. mutans* strains were made resistant to fluoride by exposure to ultraviolet light or acriflavin [49]. However, this method became less popular because of the non-specific nature of these mutagens and the latent chromosome lesions [52].

Reports on clinically isolated fluoride-resistant *S. mutans* strains are scarce. Streckfuss et al. reported seven fluoride-resistant *S. mutans* isolates from radiation-induced xerostomia patients receiving daily topical application of 1% (238 mM) NaF gel [15]. These isolates were obtained with the one-step selection method. The isolates that were able to grow in culture media containing 400–600 ppm (21.1–31.6 mM) of fluoride were considered ‘fluoride resistant’ [15]. The same research group later found that sustained fluoride treatment increased the ratio of fluoride-resistant to fluoride-sensitive strains [16]. These two studies are the only reports on the isolation of fluoride-resistant strains from clinical samples. So far, there have been no reports on the prevalence of naturally occurring fluoride-resistant bacteria in the oral cavity. The reasons for the lack of this type of studies are unknown. It might be related to the small attention that the antimicrobial function of fluoride has received, as fluoride has mainly been studied for its role in protecting dental hard tissue.

## Characteristics of fluoride-resistant strains

One of the foremost concerns related to fluoride resistance in oral bacteria is whether fluoride-resistant strains impose risks on oral health. Studies have been focused on several characteristics of fluoride-resistant strains: stability of the resistance, the acidogenicity, the fitness, and the *in vivo* cariogenicity. A summary of the fluoride-resistant *S. mutans* strains and their characteristics is shown in the Table 1.

**Table 1.** Fluoride-resistant strains and their characterizations

Fluoride-resistant *S. mutans* strain	Lab/clinical isolate	Level of resistance	Stable/transient resistance	Characteristic	Potential mechanism	Reference
1144/600FR	Lab isolate; stepwise derived from *S. mutans* #1144	600 ppm (31.6 mM)	Stable	N/A	Potential involvement of genotypic mutation.	[15]
6715/600FR	Lab isolate; stepwise derived from *S. mutans* 6715	600 ppm (31.6 mM)	Stable	Decreased ability to form biofilms on tooth compared to the wild type in the absence of fluoride;	Potential involvement of genotypic mutation.	[15]
				Higher adherence percentage than the wild type with 600 ppm (31.6 mM) fluoride		
6715-derived	Lab isolate; induced by exposure of *S. mutans* 6715 to ultraviolet light	600 ppm (31.6 mM)	N/A	Lower cariogenicity than the wild type *in vivo* (rats)	N/A	[49]
C180-2FR	Lab isolate; one-step derived from *S. mutans* C180-2	500 ppm (26.3 mM)	Stable	Faster acid production than the wild type when pH <6.0 (in the absence of fluoride);	Similar activity of enolase or F-ATPase compared to the wild type;	[17,28,53–55]
					No mutation in the enolase gene;	
				Unable to bind to enamel when the wild type was present;	Eight mutations found in C180-2FR genome; mutation *mutp* (−44A→C) upregulates the expression of fluoride exporters	
				Similar cariogenicity compared to the wild type *in vivo* (rats)		
FA-1-derived	Lab isolate; induced by exposure of *S. mutans* FA-1 to acriflavin	600 ppm (31.6 mM)	N/A	Higher cariogenicity than the wild type *in vivo* (rats)	N/A	[49]
GS-5 A25–A73	Lab isolates; stepwise derived from *S. mutans* GS-5	400–3,000 ppm (21.1–157.9 mM)	Stable	N/A	Potential involvement of multiple gene mutations.	[13]
NCH105	Lab isolate; one-step derived from *S. mutans* UA130	1,000 ppm (52.6 mM)	Stable	Higher acid production and glucose uptake than the wild type with fluoride present at pH 6.5 and pH 6.0	Higher F-ATPase ability than the wild type in the presence of fluoride	[18,47]
					Stronger enolase activity than the wild type in the presence of fluoride	
				Similar ability with the wild type to bind to artificial tooth pellicles	Two mutations found in enolase gene	
S-126-derived	Lab isolate; induced by exposure of *S. mutans* S-126 to ultraviolet light	600 ppm (31.6 mM)	N/A	Lower cariogenicity than the wild type *in vivo* (rats)	N/A	[49]
U41, U48, U53, U60, U64, U80, U85	Clinical isolates	600 ppm (31.6 mM)	Transient	Similar to adherence patterns of lab-derived fluoride-resistant strains	Potential involvement of horizontal plasmid transfer	[15]
UA159FR	Lab isolate; stepwise derived from *S. mutans* UA159	1,000 ppm (52.6 mM)	N/A	Higher ability to resist acid stress than the wild type	Mutation in *mutp* upregulates the expression of fluoride exporters	[51] and the authors’ unpublished data

The stability, or persistence, of fluoride resistance is studied as an indication of whether the fluoride-resistant strains can prosper in the oral cavity in the long run. Resistance to fluoride induced *in vitro* is usually stable and remains at similar resistance levels after as many as 500 transfers in the absence of fluoride [11,15]. However, the *S. mutans* isolates from xerostomia patients with long-term fluoride application seem to have a transient resistance, as the resistance was lost after seven transfers in fluoride-free medium [15]. Very little has been done to characterize these transient fluoride-resistant isolates. The characterizations discussed below are all based on the stable fluoride-resistant strains.

The acidogenicity of fluoride-resistant *S. mutans* strains has been studied in comparison with the corresponding wild-type fluoride-sensitive strain. The outcome of the comparison depended on the presence/absence of fluoride. When fluoride was absent, some fluoride-resistant strains produced acid at a lower rate than the wild-type strains at the environmental pH between 4.0 and 7.0 [56]. One fluoride-resistant strain was reported to produce acid at a higher rate than the wild-type strain when the environmental pH was <6.0 [53]. Another fluoride-resistant strain was found to produce acid at the same rate as the wild-type strain [47]. However, when fluoride was present, all fluoride-resistant strains were more acidogenic than the fluoride-sensitive strains [47,53,57].

The fitness of fluoride-resistant strains was assessed in two ways: the ability to survive acidic pH and to compete with the wild-type strains to adhere to tooth surfaces. One fluoride-resistant *S. mutans* strain was reported more sensitive to acid killing [54], while another fluoride-resistant strain exhibited stronger acid tolerance than its parental strain [51]. Controversial results have also been reported for the competition test, which examined the competition between fluoride-resistant and fluoride-sensitive strains to bind to enamel. A fluoride-resistant strain was unable to bind to teeth when its wild-type strain was present [55,58]. This result was challenged by Hoelscher, who found another fluoride-resistant strain to be capable of binding to the same extent as the wild-type strain when they had been mixed in equal proportions [47]. The mechanism of this competition is not very clear. Likely, the growth rate of each strain determines the outcome of the competition. In the former study [55,58], the growth rate of the fluoride-resistant strain was reported to be lower than its wild-type strain, while the strains in the latter study exhibited similar generation time [47].

A few *in vivo* studies examined the cariogenicity of fluoride-resistant *S. mutans* strains in rats. The results vary, depending on the strains and the parameters used in the experiment. Van Loveren (1989) reported less severe dentinal lesions in rats superinfected with a fluoride-resistant strain than in rats superinfected with the wild-type strain [50]. This result was in line with the results of the competition test, in which the same strains were examined [55,58]. However, when the number of all visible lesions, including enamel and dentine lesions, were taken into account, the two strains showed similar cariogenic potentials, irrespective of whether fluoride was included in the diet [50]. Rosen [1978] also reported lower cariogenicity of three fluoride-resistant strains when compared to their wild-type strains [49]. Another strain, made resistant to fluoride by exposure to ultraviolet light, exhibited at least as much ability as its wild type to cause caries [49]. As no conclusions can be drawn from these *in vivo* studies, due to their different experimental designs as well as their limited sample sizes, the cariogenicity of fluoride-resistant strains *in vivo* remains an important issue for further studies.

## Mechanisms of fluoride resistance

As previously stated, *S. mutans* can acquire either transient or stable fluoride resistance. The former was noticed in xerostomia patients with daily topical application of fluoride [15,16]. It was proposed that this transient resistance was related to the horizontal transfer of plasmids [15,16]. Fluoride-resistant *S. mutans* strains lost these plasmids when fluoride was absent and rapidly reversed back to their fluoride-sensitive state [15,16]. There is currently no evidence to support this hypothesis.

In contrast to transient fluoride resistance, stable fluoride resistance is believed to be due to chromosomal mutations. Different approaches have been applied to identify genes that are related to the stable fluoride resistance. Until 2008, the research focus has been on enolase and F-ATPase, which are known to be essential in the antimicrobial action of fluoride. In 2012, a fluoride exporter and its regulation were unexpectedly discovered in bacteria during a study on the binding of various metabolites to bacterial RNA [59,60]. A few years later, multiple gene mutations were reported in a fluoride-resistant strain [17]. These recent studies brought new concepts or candidates for the mechanism of fluoride resistance. Figure 1 shows not only the antimicrobial target sites of fluoride, but also the potential sites that are involved in fluoride resistance.

## Enolase and F-ATPase

Enolase and F-ATPase are two important enzymes that are sensitive to fluoride. Hence, these two enzymes were originally considered as the most possible sites involved in fluoride resistance. Meanwhile, enolase and F-ATPase were also thought to be involved in the mechanisms of fluoride resistance. It was hypothesized that they were insensitive to fluoride in fluoride-resistant *S. mutans* strains. In the presence of fluoride, the cellular level of PEP could be maintained, which provided sufficient substrates for the PEP-dependent PTS, as well as for glycolysis [47].

However, the published evidence is not sufficient to prove the above hypothesis. The activities of purified enolase or enolase in permeabilized cells were compared *in vitro* between fluoride-sensitive strain and the derived fluoride-resistant strains. No difference was observed between the enolase activities for the two strains, regardless the presence of fluoride [12,28]. Recently, a study reported that enolase from a fluoride-resistant strain was less sensitive to fluoride. However, the difference was not large enough to explain the resistance in metabolism [18].

The results of studies on the F-ATPase activity in fluoride-resistant strains were also inconsistent. One study showed that F-ATPase in a fluoride-resistant strain was insensitive to fluoride at pH 5.0, while the F-ATPase in the corresponding fluoride-sensitive wild-type strain was sensitive to fluoride under the same pH condition [47]. However, another study did not find any difference between two strains at both pH 4.0 and pH 7.0 [28].

In efforts to identify mutations responsible for fluoride resistance, researchers have sequenced genes coding for enolase and F-ATPase [18,28,61]. No mutation was found in the F-ATPase-coding gene in the fluoride-resistant strain [61]. In the enolase-coding gene (*eno*) of the fluoride-resistant strain NCH105, one mutation was located [18], while in another strain, no mutation was identified [28]. The *eno* mutation in the fluoride-resistant strain NCH105 led to an amino acid alteration from proline to leucine (P173L) [18]. However, in the three-dimensional conformation models, the mutation was not located nearby any known F^–^ binding site [18].

The reason for the inconsistent findings with enolase and F-ATPase in fluoride resistance is unclear. Since different fluoride-resistant strains were tested in different studies, it is possible that the approach (proteins or regulation pathways) employed by each strain to resist fluoride is strain-dependent.

## Fluoride exporters

In 2012, Breaker et al. discovered that two gene families, which were previously predicted to code for proteins involved in camphor resistance (*crcB*) and ClC-type ion channel protein (*eriC^F^*), have identical biochemical roles [59,60]. Both gene families encoded fluoride exporters [59,62] and are directly related to the fluoride resistance of microorganisms. The deletion of *crcB* in *E. coli* or *Candida albicans* leads to a 200- to 350-fold higher sensitivity of the mutant to fluoride compared to the wild-type strain. The resistance to fluoride can be restored by supplementing *eriC^F^* from another species, *Bacillus cereus* [59,63]. The genes *crcB* and *eriC^F^* are conserved in the bacterial kingdom. Most bacterial species harbor only *crcB* in their genome, while a few of them have only *eriC^F^* [59]. *S. mutans* has two *eriC^F^* genes in tandem with the same orientation, namely *perA* and *perB* [64]. The products of these two genes share 58% amino acid identity [17]. The involvement of these two genes in fluoride resistance has been confirmed by two gene knockout studies and a gene regulation study [54,64,65]. Both gene knockout studies found that *S. mutans* became 100-fold more sensitive to fluoride after knocking out both *eriC^F^* copies [64,65]. Differently, one of the two studies reported increased fluoride sensitivity by knocking out either of the two *eriC^F^* copies [65], while the other discovered that only the second *eriC^F^* copy was required for fluoride resistance [64]. In the gene regulation study, the introduction of a single mutation in the promoter, which constitutively upregulated *eriC^F^* expression, conferred fluoride resistance on the *S. mutans* strain [54]. It is currently not clear why *S. mutans* possesses two copies of fluoride exporter-coding genes. Breaker proposes that this might be a more recent adaptation made by the species to the bursts of extremely high fluoride concentrations delivered with oral health products [60].

Currently, the fluoride exporter is identified as a subclass of the bacterial CLC anion-transporting proteins based on its protein structure. In contrast to canonical CLCs, which are weakly selective for Cl^–^ and other monovalent anions, this fluoride exporter greatly prefers F^–^ over Cl^–^, even though F^–^ is usually strongly hydrated and difficult to develop host–guest compounds [66]. The protein has a ‘double-barrelled’ channel architecture in which two F^–^ tunnels span the membrane [67]. The narrow pores and unusual anion coordination that exploits the quadrupolar edges of conserved phenylalanine rings indicate its preference for F^–^ [67]. Unlike all other CLC transporters, which employ two-to-one stoichiometry, the fluoride exporter exchanges F^–^ with H^+^ with one-to-one stoichiometry [66,68].

The regulation of the fluoride exporter varies among different bacterial species. In *S. mutans*, the expression of the fluoride exporter is through the promoter of *perA* (homologue of *eriC^F^*). A single mutation (A→C) at the putative −35 element of the promoter of *perA* in strain *S. mutans* UA159 considerably upregulated the expression of both fluoride exporter genes *perA* and *perB*. Likely, this upregulation is achieved by the enhanced binding affinity of RNA polymerase to the mutated promoter [17,54]. It is worth mentioning that this is not the only regulation mechanism for the fluoride exporters. Many bacterial species, including those from the orders Lactobacillales and Bacillales, regulate the fluoride exporters using fluoride riboswitches [59]. Fluoride riboswitches are fluoride-binding RNA molecules, which are stabilized once bound by fluoride [69]. The fluoride-bound riboswitches can then activate expression of genes coding for the fluoride exporters [59].

## Involvement of multiple factors

Using state-of-the-art whole genome sequencing (WGS) and bioinformatics analyses, the genome sequences of *S. mutans* C180-2 and its derivative fluoride-resistant strain C180-2FR were compared [17]. In total, eight single nucleotide polymorphisms (SNPs) were identified in five protein-coding regions and two intergenic non-coding regions in strain C180-2FR. Two of eight SNPs were related to the fluoride exporters. One SNP locates in the promoter region of *perA*. Its involvement in the upregulation of the expression of fluoride exporter is discussed above. The other SNP locates in the coding region of *perB*, which leads to the change of an amino acid (I373V).

The list of identified SNPs provides us with multiple candidate genes that may be involved in fluoride resistance [17 and the authors’ unpublished data]. One interesting target is pyruvate kinase, a glycolytic enzyme converting PEP to pyruvate. Pyruvate kinase plays a central role in carbohydrate metabolism by connecting sugar uptake and glycolysis. The change of its configuration or activity can lead to a dramatic shift in metabolism [70]. Moreover, in the glycolytic pathway, pyruvate kinase locates downstream of enolase, the known fluoride-sensitive enzyme, in the cascade. Two SNPs are found in the pyruvate kinase-coding gene of strain C180-2FR. Back in 1987, Brussock and Kashket already proposed that fluoride resistance could be due to a cumulative effect of at least two gene mutations [13]. Until now, no synergistic effect of multiple genes has been identified for fluoride resistance. However, the multiple SNPs identified in the fluoride-resistant C180-2FR strain indicate this possibility.

## Conclusions

Fluoride is widely used as an anti-caries agent and has been for at least five decades. *S. mutans* and other oral bacterial species are able to develop resistance to counteract the antimicrobial effects of fluoride. Research on several fluoride-resistant strains has demonstrated that this resistance is stable and acquired through chromosomal mutations. However, the impact of fluoride-resistant strains on the oral microbial community and on the cariogenicity of dental biofilms is still unknown. Standardized experimental designs and protocols are essential for a better comparison between study results, which can improve the risk assessment of fluoride-resistant strains. Furthermore, profound knowledge on the mechanisms of fluoride resistance may provide us with novel molecular tools to study the prevalence of fluoride resistance in the oral microbial community.

## References

[1] Anusavice KJ, Zhang N-Z, Shen C. Effect of CaF_2_ content on rate of fluoride release from filled resins. J Dent Res. 2005;84:440–9. 15840780 10.1177/154405910508400508

[2] Lennon MA One in a million: the first community trial of water fluoridation. Bull World Health Organ. 2006;84:759–760. 17128347 10.2471/blt.05.028209 PMC2627472

[3] Buzalaf MA, Pessan JP, Honorio HM, et al Mechanisms of action of fluoride for caries control. Monogr Oral Sci. 2011;22:97–114. 21701194 10.1159/000325151

[4] Jacobson A, Strang R, Stephen K Effect of low fluoride levels in de/remineralization solutions of pH-cycling model. Caries Res. 1991;25:230–231.

[5] Van Loveren C Antimicrobial activity of fluoride and its *in vivo* importance: identification of research questions. Caries Res. 2001;35 Suppl 1:65–70. 11359062 10.1159/000049114

[6] Loesche WJ, Syed SA, Murray RJ, et al Effect of topical acidulated phosphate fluoride on percentage of *Streptococcus mutans* and *Streptococcus sanguis* in plaque. II. Pooled occlusal and pooled approximal samples. Caries Res. 1975;9:139–155. 1090368 10.1159/000260153

[7] Beighton D, McDougall WA The effects of fluoride on the percentage bacterial composition of dental plaque, on caries incidence, and on the *in vitro* growth of *Streptococcus mutans, Actinomyces viscosus*, and *Actinobacillus* sp. J Dent Res. 1977;56:1185–1191. 272378 10.1177/00220345770560101201

[8] Meurman JH Ultrastructure, growth, and adherence of *Streptococcus mutans* after treatment with chlorhexidine and fluoride. Caries Res. 1988;22:283–287. 3180159 10.1159/000261122

[9] Hamilton IR, Ellwood DC Effects of fluoride on carbohydrate metabolism by washed cells of *Streptococcus mutans* grown at various pH values in a chemostat. Infect Immun. 1978;19:434–442. 24590 10.1128/iai.19.2.434-442.1978 PMC414102

[10] Maltz M, Emilson CG Susceptibility of oral bacteria to various fluoride salts. J Dent Res. 1982;61:786–790. 6953115 10.1177/00220345820610062701

[11] Hamilton IR Growth characteristics of adapted and ultraviolet-induced mutants of *Streptococcus salivarius* resistant to sodium fluoride. Can J Microbiol. 1969;15:287–295. 5781755 10.1139/m69-052

[12] Bunick FJ, Kashket S Enolases from fluoride-sensitive and fluoride-resistant streptococci. Infect Immun. 1981;34:856–863. 7333671 10.1128/iai.34.3.856-863.1981 PMC350948

[13] Brussock SM, Kral TA Effects of pH on expression of sodium fluoride resistance in *Streptococcus mutans*. J Dent Res. 1987;66:1594–1596. 3476560 10.1177/00220345870660101701

[14] van Loveren C, Buijs JF, Ten Cate JM Protective effect of topically applied fluoride in relation to fluoride sensitivity of mutans streptococci. J Dent Res. 1993;72:1184–1190. 8360360 10.1177/00220345930720080401

[15] Streckfuss JL, Perkins D, Horton IM, et al Fluoride resistance and adherence of selected strains of *Streptococcus mutans* to smooth surfaces after exposure to fluoride. J Dent Res. 1980;59:151–158. 6928001 10.1177/00220345800590021501

[16] Brown LR, White JO, Horton IM, et al Effect of continuous fluoride gel use on plaque fluoride retention and microbial activity. J Dent Res. 1983;62:746–751. 6574158 10.1177/00220345830620061201

[17] Liao Y, Chen J, Brandt BW, et al Identification and functional analysis of genome mutations in a fluoride-resistant *Streptococcus mutans* strain. Plos One. 2015;10:e0122630. 25856576 10.1371/journal.pone.0122630 PMC4391945

[18] Mitsuhata C, Puteri MM, Ohara Y, et al Possible involvement of enolase in fluoride resistance in *Streptococcus mutans*. Pediatr Dental J. 2014;24:12–16.

[19] Loesche WJ Role of *Streptococcus mutans* in human dental decay. Microbiol Rev. 1986;50:353–380. 3540569 10.1128/mr.50.4.353-380.1986 PMC373078

[20] Hamilton IR Biochemical effects of fluoride on oral bacteria. J Dent Res. 1990;69 Spec No:660–667; discussion 82-83. 10.1177/00220345900690S128 2179327

[21] Marquis RE, Clock SA, Mota-Meira M Fluoride and organic weak acids as modulators of microbial physiology. FEMS Microbiol Rev. 2003;26:493–510. 12586392 10.1111/j.1574-6976.2003.tb00627.x

[22] Rölla G, Melsen B Desorption of protein and bacteria from hydroxyapatite by fluoride and monofluorophosphate. Caries Res. 1975;9:66–73. 1054294 10.1159/000260144

[23] Marquis RE Diminished acid tolerance of plaque bacteria caused by fluoride. J Dent Res. 1990;69 Spec No:672–675; discussion 82-83. 10.1177/00220345900690S130 2138181

[24] Bender GR, Thibodeau EA, Marquis RE Reduction of acidurance of streptococcal growth and glycolysis by fluoride and gramicidin. J Dent Res. 1985;64:90–95. 2579114 10.1177/00220345850640021701

[25] Belli WA, Buckley DH, Marquis RE Weak acid effects and fluoride inhibition of glycolysis by *Streptococcus mutans* GS-5. Can J Microbiol. 1995;41:785–791. 7585355 10.1139/m95-108

[26] Guha-Chowdhury N, Clark AG, Sissons CH Inhibition of purified enolases from oral bacteria by fluoride. Oral Microbiol Immunol. 1997;12:91–97. 9227132 10.1111/j.1399-302x.1997.tb00623.x

[27] Curran TM, Buckley DH, Marquis RE Quasi-irreversible inhibition of enolase of *Streptococcus mutans* by fluoride. FEMS Microbiol Lett. 1994;119:283–288. 8050711 10.1111/j.1574-6968.1994.tb06902.x

[28] van Loveren C, Hoogenkamp MA, Deng DM, et al Effects of different kinds of fluorides on enolase and ATPase activity of a fluoride-sensitive and fluoride-resistant *Streptococcus mutans* strain. Caries Res. 2008;42:429–434. 18832829 10.1159/000159606

[29] Germaine GR, Tellefson LM Role of the cell membrane in pH-dependent fluoride inhibition of glucose uptake by *Streptococcus mutans*. Antimicrob Agents Chemother. 1986;29:58–61. 3729335 10.1128/aac.29.1.58 PMC180364

[30] Matsui R, Cvitkovitch D Acid tolerance mechanisms utilized by *Streptococcus mutans*. Future Microbiol. 2010;5:403–417. 20210551 10.2217/fmb.09.129 PMC2937171

[31] Jensen ME, Wefel JS Human plaque pH responses to meals and the effects of chewing gum. Br Dent J. 1989;167:204–208. 2789898 10.1038/sj.bdj.4806971

[32] Welin-Neilands J, Svensater G Acid tolerance of biofilm cells of *Streptococcus mutans*. Appl Environ Microbiol. 2007;73:5633–5638. 17630302 10.1128/AEM.01049-07 PMC2042095

[33] Bender GR, Sutton SV, Marquis RE Acid tolerance, proton permeabilities, and membrane ATPases of oral streptococci. Infect Immun. 1986;53:331–338. 3015800 10.1128/iai.53.2.331-338.1986 PMC260879

[34] Sutton SV, Bender GR, Marquis RE Fluoride inhibition of proton-translocating ATPases of oral bacteria. Infect Immun. 1987;55:2597–2603. 2889674 10.1128/iai.55.11.2597-2603.1987 PMC259948

[35] Pandit S, Kim H-J, Song K-Y, et al. Relationship between fluoride concentration and activity against virulence factors and viability of a cariogenic biofilm: *in vitro* study. Caries Res. 2013;47:539–547. 23774608 10.1159/000348519

[36] Marquis RE Antimicrobial actions of fluoride for oral bacteria. Can J Microbiol. 1995;41:955–964. 7497353 10.1139/m95-133

[37] Duncan TM, Duser MG, Heitkamp T, et al Regulatory conformational changes of the epsilon subunit in single FRET-labeled FoF1-ATP synthase. Proc SPIE Int Soc Opt Eng. 2014;8948:89481J. 10.1117/12.2040463 PMC4112770 25076824

[38] Johnson KM, Swenson L, Opipari AW Jr., et al Mechanistic basis for differential inhibition of the F_1_F_o_-ATPase by aurovertin. Biopolymers. 2009;91:830–840. 19462418 10.1002/bip.21262 PMC2757082

[39] Shani S, Friedman M, Steinberg D The anticariogenic effect of amine fluorides on *Streptococcus sobrinus* and glucosyltransferase in biofilms. Caries Res. 2000;34:260–267. 10867426 10.1159/000016600

[40] Schilling K, Blitzer M, Bowen W Adherence of *Streptococcus-mutans* to glucans formed *in situ* in salivary pellicle. J Dent Res. 1989;68:1678–1680.

[41] Pandit S, Kim JE, Jung KH, et al Effect of sodium fluoride on the virulence factors and composition of *Streptococcus mutans* biofilms. Arch Oral Biol. 2011;56:643–649. 21241981 10.1016/j.archoralbio.2010.12.012

[42] Guo L, Ying Y, Hong X, et al A Preliminary study of exogenous dextranase and NaF directly influence *Streptococcus mutans* glucosyltransferase activity. J Pure Appl Microbiol. 2014;8:21–28.

[43] Burne RA, Marquis RE Alkali production by oral bacteria and protection against dental caries. FEMS Microbiol Lett. 2000;193:1–6. 11094270 10.1111/j.1574-6968.2000.tb09393.x

[44] Clancy A, Burne RA Construction and characterization of a recombinant ureolytic *Streptococcus mutans* and its use to demonstrate the relationship of urease activity to pH modulating capacity. FEMS Microbiol Lett. 1997;151:205–211. 9228755 10.1111/j.1574-6968.1997.tb12571.x

[45] Curran TM, Ma Y, Rutherford GC, et al Turning on and turning off the arginine deiminase system in oral streptococci. Can J Microbiol. 1998;44:1078–1085. 10030002 10.1139/cjm-44-11-1078

[46] Parfenyev AN, Salminen A, Halonen P, et al Quaternary structure and metal ion requirement of family II pyrophosphatases from *Bacillus subtilis, Streptococcus gordonii*, and *Streptococcus mutans*. J Biol Chem. 2001;276:24511–24518. 11342544 10.1074/jbc.M101829200

[47] Hoelscher GL, Hudson MC Characterization of an unusual fluoride-resistant *Streptococcus mutans* isolate. Curr Microbiol. 1996;32:156–161. 8704659 10.1007/s002849900028

[48] Lau KA, Kral TA Isolation and characterization of low-pH fluoride-resistant mutants of *Streptococcus mutans*. Oral Microbiol Immunol. 1987;2:136–138. 3507624 10.1111/j.1399-302x.1987.tb00278.x

[49] Rosen S, Frea JI, Hsu SM Effect of fluoride-resistant microorganisms on dental caries. J Dent Res. 1978;57:180. 277510 10.1177/00220345780570020301

[50] van Loveren C, Lammens AJ, Ten Cate JM *In vitro* induced fluoride resistance of *Streptococcus mutans* and dental caries in rats. Caries Res. 1989;23:358–364. 2766322 10.1159/000261207

[51] Zhu L, Zhang Z, Liang J Fatty-acid profiles and expression of the *fabM* gene in a fluoride-resistant strain of *Streptococcus mutans*. Arch Oral Biol. 2012;57:10–14. 21741617 10.1016/j.archoralbio.2011.06.011

[52] Auerbach C Mutation research: problems, results and perspectives. New York: Springer; 2013.

[53] Van Loveren C, Van de Plassche-Simons YM, De Soet JJ, et al Acidogenesis in relation to fluoride resistance of *Streptococcus mutans*. Oral Microbiol Immunol. 1991;6:288–291. 1820566 10.1111/j.1399-302x.1991.tb00494.x

[54] Liao Y, Brandt BW, Zhang M, et al A single nucleotide change in the promoter *mutp* enhances fluoride resistance of *Streptococcus mutans*. Antimicrob Agents Chemother. 2016;60:7509–7512. 27697763 10.1128/AAC.01366-16 PMC5119038

[55] van Loveren C, Lammens AJ, Ten Cate JM Development and establishment of fluoride-resistant strains of *Streptococcus mutans* in rats. Caries Res. 1990;24:337–343. 2148122 10.1159/000261293

[56] Eisenberg AD, Wegman MR, Oldershaw MD, et al Effect of fluoride, lithium or strontium on acid production by pelleted human dental plaque. Caries Res. 1985;19:454–457. 3864546 10.1159/000260881

[57] Sheng JY, Liu Z Acidogenicity and acidurance of fluoride-resistant *Streptococcus sobrinus in vitro*. Chin J Dent Res. 2000;3:7–14. 11314523

[58] van Loveren C, Buys JF, de Soet JJ, et al Competition between fluoride-resistant and fluoride-sensitive *Streptococcus mutans* in rat dental plaque. Caries Res. 1991;25:424–430. 1810654 10.1159/000261405

[59] Baker JL, Sudarsan N, Weinberg Z, et al Widespread genetic switches and toxicity resistance proteins for fluoride. Science. 2012;335:233–235. 22194412 10.1126/science.1215063 PMC4140402

[60] Breaker RR New insight on the response of bacteria to fluoride. Caries Res. 2012;46:78–81. 22327376 10.1159/000336397 PMC3331882

[61] Sheng JY, Huang ZW, Liu Z A comparison of the activities of membrane-bound, proton translocating ATPases between *Streptococcus mutans* fluoride-resistant and their parent strains. Shanghai Kou Qiang Yi Xue. 2005;14:71–73. 15747020

[62] Rapp M, Granseth E, Seppala S, et al Identification and evolution of dual-topology membrane proteins. Nat Struct Mol Biol. 2006;13:112–116. 16429150 10.1038/nsmb1057

[63] Li S, Smith KD, Davis JH, et al Eukaryotic resistance to fluoride toxicity mediated by a widespread family of fluoride export proteins. Proc Natl Acad Sci U S A. 2013;110:19018–19023. 24173035 10.1073/pnas.1310439110 PMC3839697

[64] Men X, Shibata Y, Takeshita T, et al Identification of anion channels responsible for fluoride resistance in oral streptococci. Plos One. 2016;11:e0165900. 27824896 10.1371/journal.pone.0165900 PMC5100911

[65] Murata T, Hanada N Contribution of chloride channel permease to fluoride resistance in *Streptococcus mutans*. FEMS Microbiol Lett. 2016;363:fnw101. 27190286 10.1093/femsle/fnw101

[66] Stockbridge RB, Lim HH, Otten R, et al Fluoride resistance and transport by riboswitch-controlled CLC antiporters. Proc Natl Acad Sci U S A. 2012;109:15289–15294. 22949689 10.1073/pnas.1210896109 PMC3458365

[67] Stockbridge RB, Kolmakova-Partensky L, Shane T, et al Crystal structures of a double-barrelled fluoride ion channel. Nature. 2015;525:548–551. 26344196 10.1038/nature14981 PMC4876929

[68] Picollo A, Xu Y, Johner N, et al Synergistic substrate binding determines the stoichiometry of transport of a prokaryotic H(+)/Cl(-) exchanger. Nat Struct Mol Biol. 2012;19:525–531, S1. 22484316 10.1038/nsmb.2277 PMC3348462

[69] Ames TD, Rodionov DA, Weinberg Z, et al A eubacterial riboswitch class that senses the coenzyme tetrahydrofolate. Chem Biol. 2010;17:681–685. 20659680 10.1016/j.chembiol.2010.05.020 PMC3417113

[70] Christofk HR, Vander Heiden MG, Harris MH, et al The M2 splice isoform of pyruvate kinase is important for cancer metabolism and tumour growth. Nature. 2008;452:230–233. 18337823 10.1038/nature06734

